# Alpha-L-Fucosidase Serves as a Prognostic Indicator for Intrahepatic Cholangiocarcinoma and Inhibits Its Invasion Capacity

**DOI:** 10.1155/2018/8182575

**Published:** 2018-02-22

**Authors:** Zeyu Shuang, Yize Mao, Guohe Lin, Jun Wang, Xin Huang, Jianlin Chen, Fangting Duan, Shengping Li

**Affiliations:** ^1^State Key Laboratory of Oncology in South China, Collaborative Innovation Center for Cancer Medicine, Sun Yat-sen University Cancer Center, Guangzhou 510060, China; ^2^Department of Breast Oncology, Sun Yat-sen University Cancer Center, Guangzhou 510060, China; ^3^Department of Hepatobiliary Oncology, Sun Yat-sen University Cancer Center, Guangzhou 510060, China; ^4^Department of Oncology, Second Affiliated Hospital of Anhui Medical University, Anhui 230000, China; ^5^Department of Experimental Research, Sun Yat-sen University Cancer Center, Guangzhou 510060, China

## Abstract

Alpha-L-fucosidase (AFU) has been reported to be a predictor of survival in patients with several cancers, but it is unclear whether AFU is associated with prognosis in patients with intrahepatic cholangiocarcinoma (iCCA). In this study, we used receiver operating characteristic (ROC) analysis to generate the cutoff point of AFU for overall survival (OS). The prognostic influence of the AFU level in serum on OS was studied using Kaplan-Meier curves. Moreover, invasion assays and Western blotting were performed to explore the effects of AFU on iCCA invasion in vitro. We found that higher AFU levels (≥20.85 U/L) were significantly associated with favorable median OS (44.3 months versus 20.1 months; *P* = 0.022) in iCCA patients. Cox regression models' analyses showed that the AFU level was an independent predictor for OS (*P* = 0.006). Moreover, our results revealed that the AFU could impair the invasion capability of the iCCA cells, HuH28, and also downregulated the expression of matrix metalloproteinase 2 and matrix metalloproteinase 9. In conclusion, our results indicate that AFU is a significantly favorable prognostic factor in iCCA patients.

## 1. Introduction

Intrahepatic cholangiocarcinoma (iCCA) is a rare but aggressive malignancy arising from the epithelium of biliary ducts [1], accounting for 5% to 30% of all primary liver malignancies [2–5]. Complete surgical resection was believed to be the best hope for cure [6]. However, even if patients are eligible for curative hepatectomy, the prognosis of iCCA is poor, with a 5-year survival rate of 22% to 31% [6, 7]. Therefore, it is important to highlight predictive factors in patients with iCCA, which may help to guide appropriate clinical management and prolong patients' survival time.

Alpha-L-fucosidase (AFU), a liposomal enzyme that participates in the degradation of various fucose-containing fucoglycoconjugates has been used as a tumor marker in the diagnosis of hepatic carcinoma and colorectal cancer [8, 9]. A recent study showed that high AFU levels in serum were associated with poor outcomes in hepatocellular carcinoma [10], having a negative effect on the prognosis of patients with this type of cancer. However, possibly due to tumor heterogeneity, some studies have shown that higher AFU levels were associated with better outcomes in breast cancer [11, 12]. Higher invasion capacity is usually a cause of poor prognosis in cancer [13].

Matrix metalloproteinase 9 (MMP-9), an enzyme that degrades collagen IV to destroy basement membrane, has been shown to promote tumor invasion [14]. A recent study showed that AFU decreased the invasion of human breast cancer cells by downregulating MMP-9, which may partially explain the correlation between lower levels of AFU and poor prognosis in breast cancer [11]. As described above, AFU levels have shown different effects on outcome in different cancers; however, the prognostic significance of serum AFU levels has not so far been explored in iCCA.

In this study, we determined the best cutoff value for the preoperative AFU level and evaluated the association of AFU level with clinical outcome in patients with iCCA. In addition, we also explored the function of AFU on the invasion capacity of an iCCA cell line.

## 2. Materials and Methods

### 2.1. Study Population

This retrospective study was conducted on a primary cohort of the patients with histologically confirmed iCCA. All patients underwent surgery between August 1, 1999, and August 1, 2014, in the Sun Yat-sen University Cancer Center (Guangdong, China). Follow-up evaluations were performed every 3 months during the first 5 years and annually thereafter. This retrospective study was approved by the Institutional Review Board (IRB) of the Sun Yat-sen University Cancer Center.

### 2.2. Clinical Data Collection

All of the clinical and pathological information was collected from medical records at the Sun Yat-sen University Cancer Center. Clinicopathological data included age, sex, lymph node metastasis, tumor number, tumor size, and TNM stage. The tumor stage was determined according to the 7th TNM staging system established by the Union for International Cancer Control and the American Joint Committee on Cancer (AJCC) [15]. Laboratory data including ALT, AST, AFU, and CA19-9 were collected from the preoperative examinations. The serum AFU activity was detected by 7600 Clinical Analyzer obtained from Hitachi High-Technologies (Tokyo, Japan) as previously described [16]. Overall survival (OS) was defined as the time (in months) between the date of surgery and the date of the death.

### 2.3. Cell Culture

The human iCCA cell line HuH28 was obtained from RIKEN (Saitama, Japan), maintained in a 37°C humidified incubator, and cultured in Roswell Park Memorial Institute (RPMI) 1640 (Invitrogen Corp., USA) supplemented with 10% heat-inactivated fetal bovine serum (Gibco-BRL, Carlsbad, California, USA).

### 2.4. AFU Treatment

AFU (Sigma-Aldrich, St. Louis, Missouri, USA) was diluted in sterile phosphate-buffered saline (PBS) to a concentration of 1.69 mU/mL (8.8 mU/10^6^ cells) as described previously [11]. After being mixed with AFU (8.8 mU AFU/10^6^ cells), the HuH28 cell line was incubated at 37°C for 30 minutes. In parallel, the same number of cells was treated with PBS or AFU plus 1 nM deoxyfuconojirimycin (DFJ; Enzo Life Sciences, New York, New York, USA) and simultaneously incubated at 37°C for 30 minutes. Cells were finally washed with PBS and centrifuged to remove any residual AFU or DFJ.

### 2.5. Protein Extraction, Western Blot, and Antibodies

Following treatment with PBS/AFU/AFU + DFJ for 30 minutes as described above, protein was extracted from the HuH28 cells. Protein lysates were prepared with radioimmunoprecipitation assay (RIPA) buffer (Cell Signaling Technology, Danvers, USA) supplemented with 1 mM of phenylmethanesulfonyl fluoride (Sigma-Aldrich, USA) as recommended.

For the Western blot, a volume of extract equivalent to 15 *μ*g of total protein was run in each lane. The antibodies used were antibody against MMP-9 (Cell Signaling Technology; 1 : 500), antibody against MMP-2 (Merck Millipore, Bedford, USA; 1 : 500), and antibody against *α*-tubulin (Santa Cruz Biotechnology, Santa Cruz, USA; 1 : 5000).

### 2.6. Cell Invasion Assay

The invasion assays were conducted using Matrigel Invasion chambers (8 *μ*m; Corning BioCoat, Cambridge, Massachusetts, USA) according to the manufacturer's instructions. Briefly, after being treated with PBS/AFU/AFU + DFJ for 30 minutes as described above, the cells were resuspended in serum-free medium to a final concentration of 7 × 10^5^/mL. The cell suspension (200 *μ*L) was then pipetted into the top chamber. Medium (800 *μ*L) with 10% fetal bovine serum was added to the lower chamber as a chemoattractant. After a 24-hour incubation, the cells on the upper side of the membrane were mechanically removed with cotton swabs, and cells that had migrated to the lower surface were fixed with 100% methanol and stained with 0.1% crystal violet. The cells were counted in five fields of triplicate membranes at ×100 magnification using an Olympus IX71 microscope.

### 2.7. Cell Viability

To assess cell viability, cells were trypsinized, resuspended in PBS, and counted, before subsets were treated with PBS/AFU/AFU + DFJ as described. A volume of 100 *μ*L of medium containing 2 × 10^3^ cells/well was then plated onto a 96-well culture plate. At 24 hours after treatment, a Cell Counting Kit-8 (CCK8) assay was performed. For the latter, 10 *μ*L CCK-8 solution (Dojindo, Kumamoto, Japan) was added to each well and incubated at 37°C with 5% CO_2_ for 2.5 hours. The optical density, after calibration, was read with a microplate reader (Bio-Rad, La Jolla, USA) at 450 nm. The experiments were repeated for a minimum of three times.

### 2.8. Statistical Analyses

The optimal cutoff values for AFU were determined using a receiver operating characteristic (ROC) curve. The cutoff value that was chosen was the level where the score was closest to the point with both maximum sensitivity and specificity. The AFU values were categorized into two groups: <20.85 U/L and ≥20.85 U/L.

For continuous variables, the data were expressed as mean ± standard deviation (SD) and compared by Student's *t*-test (two-sided). Categorical variables were compared using the *χ*^2^ test or Fisher's exact test where appropriate. The Cox proportional hazards model was used for univariate and multivariate analyses. By the Kaplan-Meier method, patients' clinical endpoints were calculated and compared using the log-rank test. All of the factors entered into a multivariate analysis had a *P* value < 0.05 on univariate analysis. Hazard ratios (HRs) and their corresponding 95% confidence intervals (CIs) were estimated by Cox regression analysis.

All analyses were carried out using IBM SPSS Statistics software, version 20.0 (SPSS, Inc.). *P* values < 0.05 in two-tailed tests were considered significant.

## 3. Results

### 3.1. Patients

A total of 165 patients with iCCA were enrolled, with 148 patients being included in the analysis and 17 patients being excluded for incomplete preoperative laboratory data (*n* = 10) or follow-up after surgery of <3 months (*n* = 7).

### 3.2. ROC Analysis of AFU

**Figure 1:**
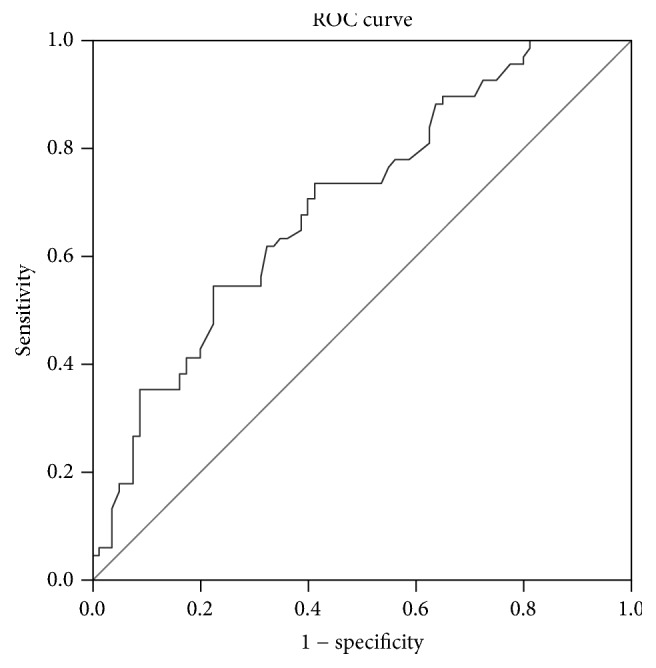
Receiver operating characteristic (ROC) analysis of the effect of serum alpha-L-fucosidase (AFU) level on overall survival. In this model, sensitivity was 73.5% and specificity was 58.8%. The area under the curve (AUC) was 0.696 (95% confidence interval (CI): 0.612–0.780; *P* < 0.001).

In the present study, we used ROC curve analysis for survival prediction to verify the optimal cutoff points for AFU (Figure 1). The results indicated that a serum AFU value of 20.85 U/L had the most significant predictive value on OS. The patients were therefore divided into two main groups using this optimal cutoff level for AFU. The clinicopathological characteristics of the patients are detailed in Table 1. There were 65 patients (43.9%) with an AFU level < 20.85 U/L and 83 patients (56.1%) with a level ≥ 20.85 U/L. The *χ*^2^ test revealed that there were no significant differences between the two groups.

### 3.3. Univariate and Multivariate Analyses of AFU as an Independent Prognostic Factor for OS

Univariate and multivariate analyses were performed to explore the significance of AFU level on the prognosis of patients with iCCA. The results of the Cox regression hazards model for predictors of OS are shown in Table 2.

Univariate analysis showed that lymph node metastasis (HR = 2.746; 95% CI = 1.744–4.323; *P* < 0.001), tumor number (HR = 1.856; 95% CI = 1.191–2.893; *P* = 0.006), tumor size (HR = 2.210; 95% CI = 1.364–3.581; *P* = 0.001), TNM stage (HR = 3.542; 95% CI = 1.983–6.328; *P* < 0.001), AFU (HR = 0.596; 95% CI = 0.381–0.932; *P* = 0.023), and CA19-9 (HR = 2.793, 95% CI = 1.786–4.368; *P* < 0.001) were associated with OS.

On multivariate analysis, lymph node metastasis (HR = 2.407; 95% CI = 1.435–4.037; *P* = 0.001), AFU (HR = 0.526; 95% CI = 0.331–0.834; *P* = 0.006), TNM stage (HR = 2.677; 95% CI = 1.418–5.053; *P* = 0.002), and CA19-9 (HR = 2.778, 95% CI = 1.748–4.412; *P* < 0.001) were predictors of OS.

**Figure 2:**
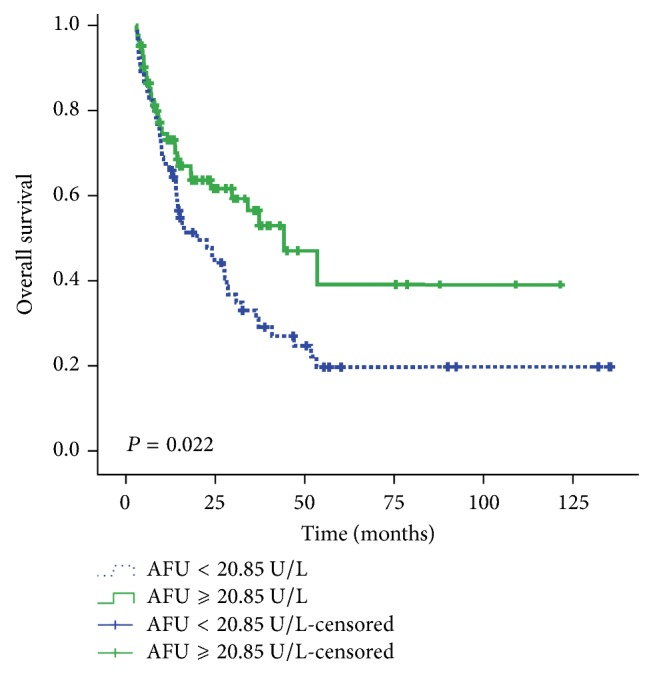
Kaplan-Meier plots of overall survival (OS) among 148 patients with intrahepatic cholangiocarcinoma (iCCA). Patients with a high level (≥20.85 U/L) of alpha-L-fucosidase (AFU; green curve) had a better prognosis than patients with low level (<20.85 U/L) of AFU (blue curve; log-rank, *P* = 0.022).

In summary, on univariate analysis, an elevated preoperative level of AFU was significantly associated with prolonged OS and remained significant in the multivariate analysis. Moreover, patients with an AFU level of <20.85 U/L showed a median OS of 20.1 months, whereas patients with an AFU level of ≥20.85 U/L had a median OS of 44.3 months (*P* = 0.022; Figure 2).

### 3.4. AFU Inhibited Invasion in iCCA Cells

A higher level of AFU in patients with iCCA was associated with better overall survival. As tumor invasion is critical to the metastasis of a cancer, which often ends in the death of the patient [13], we postulated that a high level of AFU in iCCA cells might impede the metastatic features of iCCA cells. To confirm this hypothesis, we performed invasion assays in HuH28 cells with AFU and AFU + DFJ/PBS as controls.

**Figure 3:**
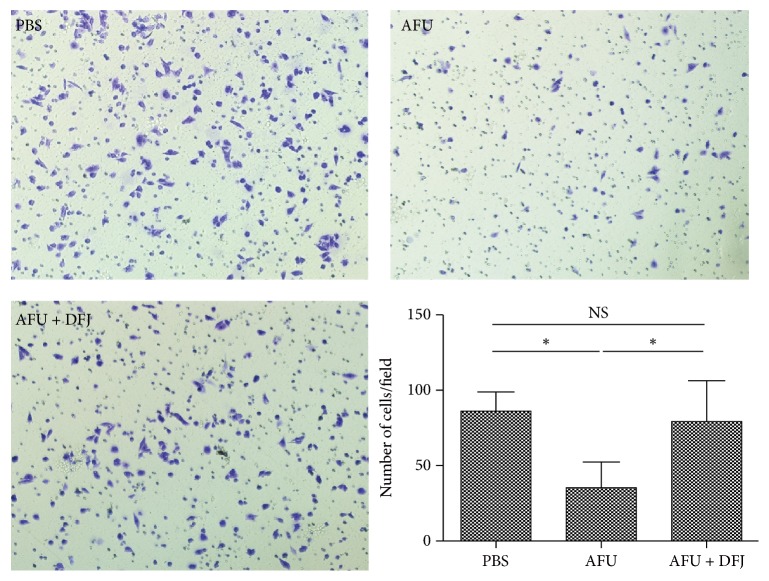
Invasion assays were used to detect the motility of HuH28 cells treated with phosphate-buffered saline (PBS)/alpha-L-fucosidase (AFU)/AFU + deoxyfuconojirimycin (DFJ) for 30 minutes. The cells that invaded or migrated to the lower side were counted using a microscope. Original magnification of images shown: ×100. Differences in invasion between the groups were analyzed by the Mann–Whitney test. ^*∗*^*P* < 0.05.

As expected, exogenous AFU decreased the invasion ability of iCCA cells, as indicated by the decreased number of migrated cells (Figure 3), but this effect of AFU was almost completely blocked by DFJ. Moreover, to exclude interference from the AFU on the number of cells, we performed a CCK8 assay. Our results showed that AFU did not inhibit proliferation of the HuH28 cells (Supplementary Figure  1). These results suggest that AFU may weaken the invasive abilities of iCCA cells.

### 3.5. AFU Decreased the Invasion Ability of iCCA Cells by Decreasing the Expression of MMP-2 and MMP-9

**Figure 4:**
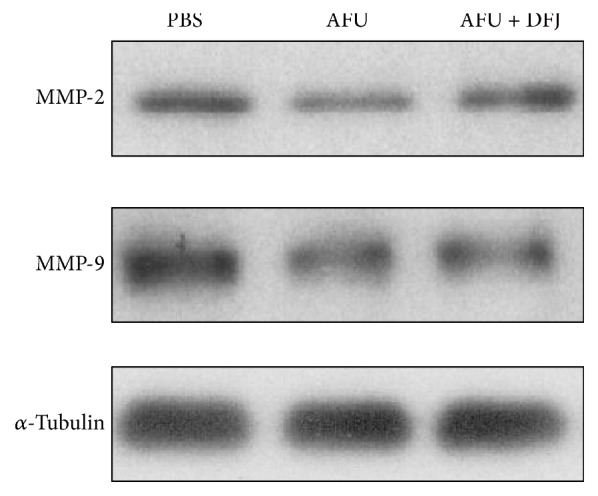
Western blotting assays for matrix metalloproteinase 2 (MMP-2) and MMP-9 in HuH28 cells. After treatment with alpha-L-fucosidase (AFU), the expression of MMP-2 and MMP-9 in HuH28 was lower than that in the phosphate-buffered saline (PBS) control and the cells treated with AFU + deoxyfuconojirimycin (DFJ).

To explore the mechanism by which AFU inhibits the invasion of HuH28 cells, we next tested the effect of AFU on the expression of two MMPs, MMP-2 and MMP-9, which are crucial to cellular invasion [17, 18]. Western blot analysis showed that AFU significantly decreased the expression of MMP-2 and MMP-9 in HuH28 cells (Figure 4). This data showed that the AFU likely diminished the capacity of invasion in HuH28 cells by downregulating their levels of MMP-2 and MMP-9.

## 4. Discussion

Our study highlights the significance of the preoperative serum AFU level for evaluating likely OS in patients with iCCA. In this study, the AFU level was confirmed to be an independent prognostic factor in patients with iCCA. By multivariate analysis, lymph node metastasis, CA19-9, and AFU level were associated with OS in iCCA patients. Moreover, AFU was shown to decrease the invasion capability of HuH28 cells by downregulating MMP-2 and MMP-9.

AFU, a lysosomal enzyme that hydrolyzes alpha-L-fucose by cleaving *α*-1,2, *α*-1,3, *α*-1,4, and *α*-1,6 linkages in the glycosylation chains is believed to be a tumor marker in the diagnosis of hepatic carcinoma and colorectal cancer [8, 9].

A recent study has shown that a higher preoperative serum AFU level was associated with poor outcomes in hepatic carcinoma, having a negative effect on prognosis [10]. However, other studies have shown that the level of AFU was higher in normal tissue than in tumor tissue and lower AFU levels were associated with poor prognosis in patients with breast cancer [9, 12, 19]. In this research, we firstly studied the prognostic effect of the serum AFU level on OS in iCCA patients. Because various cutoff values have been used when assessing the relationship between AFU level and OS in different cancers [10, 12], we used ROC curve analyses to verify the optimal cutoff point for AFU in this study. Patients with iCCA were therefore categorized into two groups: AFU level < 20.85 and ≥20.85 U/L. Using this cutoff, we found that AFU level is an independent prognostic factor in patients with iCCA by both univariate and multivariate analyses. Our results showed that a lower AFU level was associated with a poorer prognosis in patients with iCCA.

Large numbers of factors impact on the prognosis of patients with cancer. Tumor invasion is an important factor in cancer metastasis, which often ends in the death of the patient [13]. MMPs are zinc-dependent endopeptidases, which play important roles in tumor invasion by degrading collagen IV to destroy basement membrane [14]. Furthermore, MMP-2 has been shown to enhance the capacity of cellular invasion by interaction with *α*v*β*3 integrin [20]. Similarly, MMP-9 also enhances cell migration and metastatic capacity by activating *α*v*β*3 integrin [21]. Recent research showed that MMP-2 and MMP-9 were expressed in iCCA and participated in tumor invasion and metastasis [22–24]. Moreover, it has been reported that AFU was able to decrease the activity of MMP-9, therefore diminishing the invasive capability of breast cancer [11, 25].

In this study, we explored the effect of AFU on metastasis in an iCCA cell line using an invasion assay. As expected, our results showed that AFU could indeed inhibit the metastasis of the iCCA cell line. We also found that AFU downregulated the expression of MMP-2 and MMP-9. All of these results showed that AFU could impede the metastatic features of iCCA cells, explaining possibly how it may be associated with better OS of iCCA patients.

Even though AFU is an easily measurable parameter in clinical practice, there are several limitations in the present study. First, the number of patients was relatively small and this is a retrospective, observational study, which lacks external validity. Second, numerous studies have shown different cutoff values for the AFU level, so this requires further validation. Nevertheless, our work is the first to suggest the possible usefulness of the AFU level, highlighting its prognostic role, in patients with iCCA.

In conclusion, the AFU level is an easily measurable biomarker that reflects prognosis in patients with iCCA. Preoperative AFU levels may help in predicting OS and guiding clinical management. As AFU was able to inhibit the invasion capacity of iCCA, further attempts should be made to explore the mechanism of downregulation of MMP-2 and MMP-9 induced by AFU and the best strategy involving AFU for iCCA treatment to prolong the survival of the patients with iCCA.

## Figures and Tables

**Table 1:** Relationship between clinicopathological characteristics and serum alpha-L-fucosidase (AFU) level in 148 patients with intrahepatic cholangiocarcinoma (iCCA).

Variables	Number	AFU (U/L)		*P* value
		<20.85	≥20.85	
Age (years)				
<60	91	45 (69.2)	46 (55.4)	0.087
≥60	57	20 (30.8)	37 (44.6)	
Sex				
Female	54	23 (35.4)	31 (37.3)	0.623
Male	94	42 (64.6)	52 (62.7)	
Lymph node metastasis				
No	105	47 (72.3)	58 (69.9)	0.747
Yes	43	18 (27.7)	25 (30.1)	
Tumor number				
Solitary	95	41 (63.1)	54 (65.1)	0.803
Multiple	53	24 (36.9)	29 (34.9)	
ALT (U/L)				
≤40	112	51 (78.5)	61 (73.5)	0.485
>40	36	14 (21.5)	22 (26.5)	
AST (U/L)				
≤45	132	61 (93.8)	71 (85.5)	0.106
>45	16	4 (6.2)	12 (14.5)	
Tumor size (cm)				
≤5	57	23 (35.4)	34 (41)	0.489
>5	91	42 (64.6)	49 (59)	
TNM stage				
I	46	24 (36.9)	22 (26.5)	0.174
II–IV	102	41 (63.1)	61 (73.5)	
CA19-9 (U/mL)				
<100	94	40 (61.5)	54 (65.1)	0.659
≥100	54	25 (38.5)	29 (34.9)	

AST: aspartate transaminase; ALT: alanine aminotransferase; CA19-9: carbohydrate antigen 19-9.

**Table 2:** Univariate and multivariate analyses of factors affecting overall survival in patients with intrahepatic cholangiocarcinoma (iCCA).

Characteristics	Univariate		Multivariate	
	Hazard ratio (95% CI)	*P* value	Hazard ratio (95% CI)	*P* value
Age (years)				
<60	1 (reference)	0.925	n.d.	n.d.
≥60	0.978 (0.614–1.558)			
Gender				
Female	1 (reference)	0.119	n.d.	n.d.
Male	1.464 (0.907–2.363)			
Lymph node metastasis				
No	1 (reference)	<0.001	1 (reference)	0.001
Yes	2.746 (1.744–4.323)		2.407 (1.435–4.037)	
Tumor number				
Solitary	1 (reference)	0.006		NS
Multiple	1.856 (1.191–2.893)			
Tumor size (cm)				
≤5	1 (reference)	0.001		NS
>5	2.210 (1.364–3.581)			
TNM stage				
I	1 (reference)	<0.001	1 (reference)	0.002
II–IV	3.542 (1.983–6.328)		2.677 (1.418–5.053)	
AFU (U/L)				
<20.85	1 (reference)	0.023	1 (reference)	0.006
≥20.85	0.596 (0.381–0.932)		0.526 (0.331–0.834)	
CA19-9 (U/mL)				
<100	1 (reference)	<0.001	1 (reference)	<0.001
≥100	2.793 (1.786–4.368)		2.778 (1.748–4.412)	
ALT (U/L)				
≤40	1 (reference)	0.664	n.d.	n.d.
>40	1.117 (0.678–1.839)			
AST (U/L)				
≤45	1 (reference)	0.210	n.d.	n.d.
>45	0.608 (0.280–1.323)			

CI: confidence interval; AST: aspartate transaminase; ALT: alanine aminotransferase; AFU: alpha-L-fucosidase; CA19-9: carbohydrate antigen 19-9; n.d.: not done; NS: no significance.

## Supplementary Materials

Supplementary Materials Supplementary Figure 1: the CCK8 assay was used to detect the viability of the HuH28 cell line after treatment with PBS/AFU/AFU + DFJ.
