# Supplementary material for: Alpha-L-Fucosidase Serves as a Prognostic Indicator for Intrahepatic Cholangiocarcinoma and Inhibits Its Invasion Capacity
Source: Biomed Res Int. 2018 Feb 22;2018:8182575. doi: 10.1155/2018/8182575 (PMC5842738; doi:10.1155/2018/8182575)
Supplement: Supplementary Materials — Supplementary Figure 1: the CCK8 assay was used to detect the viability of the HuH28 cell line after treatment with PBS/AFU/AFU + DFJ. [file 8182575.f1.docx]

**Supplementary Fig.1:** The Cell Counting Kit-8 (CCK8) assay was used to detect the viability of the HuH28 cell line after treatment with phosphate buffered saline (PBS)/ alpha-L-fucosidase (AFU)/ AFU + deoxyfuconojirimycin (DFJ). There were no differences among the groups.
